# OSH risk management policies between North America and Southeast Asia—a comparative review

**DOI:** 10.3389/fpubh.2025.1642941

**Published:** 2025-08-19

**Authors:** Arjun Kathayat, Mohd Rafee Baharudin, Wilson Wui Siang Lee, Mohd Zahirasri Mohd Tohir

**Affiliations:** ^1^Integrity Maintenance Ltd., Carlyle, SK, Canada; ^2^Department of Community Health, Faculty of Medicine and Health Sciences, Universiti Putra Malaysia, Serdang, Malaysia; ^3^Safety Engineering Interest Group (SEIG), Department of Chemical and Environmental Engineering, Faculty of Engineering, Universiti Putra Malaysia, Serdang, Malaysia; ^4^Faculty of Engineering, Universiti Putra Malaysia, Serdang, Malaysia

**Keywords:** occupational health and safety, policies, prescriptive, performance-based, risk management, HIRARC

## Abstract

**Introduction:**

Literature suggests that a comparative analysis of occupational safety and health (OSH) policies may provide valuable insights into creating and maintaining safer and healthier workplaces. However, there are ongoing debates about which type of OSH policies will be more effective. Furthermore, there is limited or no knowledge in the literature on the comparative analysis of OSH risk management policies between North America (Saskatchewan, Canada, and the USA) and Southeast Asia (Malaysia, Singapore, and Thailand).

**Methods:**

This review employed Preferred Reporting Items for Systematic Reviews and Meta-Analysis (PRISMA) to ensure the eligibility of included regional OSH policies and employed the Population, Intervention, Comparison, Outcome, and Study (PICOS) framework to develop search questions. This review conducted a high-level qualitative analysis to assess and compare the types of OSH policies and utilized a quantitative analysis to determine the effectiveness of these policies in the regions based on the data associated with Sustainable Development Goal (SDG) 8.1.1.

**Results:**

A qualitative analysis of regional OSH policies revealed that the North American regions adopted more performance-based OSH policy styles. In contrast, Southeast Asian regions tended to practice more prescriptive OSH policies. Singapore reported the lowest injury rates (both non-fatal and fatal) and the highest ratio of OSH inspectors to workers. General multivariate regression analysis indicated a significant and positive relationship between the ratios of OSH inspectors to employed persons and non-fatal injury rates, but the negative relationship between the OSH inspectors and fatal injury rate was neither significant nor reliable.

**Conclusion:**

The findings of this research validate the current literature. Additionally, higher ratios of OSH Inspectors to employed persons may significantly contribute to reducing regional non-fatal injuries. With larger sample sizes and primary research data, future researchers can build upon the findings of this research, including the optimal effective ratios of OSH inspectors to employed persons to prevent or minimize human suffering and loss. Practitioners may constantly monitor the effectiveness of the ratios to enhance the Sustainability Development Goal (SDG) 8.1.1 performance in the regions.

## 1 Introduction

Occupational health and safety-related policies and best practices may play constructively proactive roles to motivate the workplace parties such as business organizations, industries, and workers (1), as well as the policy enforcement agencies (2) within the governments to create and maintain more compliant, more responsible (3, 4), safer, and healthier workplaces (5–8) and communities (9, 10). However, the debate among the regional policymakers, academia, and the business world on what approach to occupational safety and health (OSH) policymaking will be more effective: the top–down public (OSH and risk management) policymaking approach, where the regional governmental bodies create the OSH policies, or the OSH policymaking approach that engages with all stakeholders and makes multiple stakeholders feel ownership (11) in the regional OSH policies, has not settled yet. Likewise, when creating or implementing OSH policies, it is important to ensure that the types of OSH policies comparatively meet or exceed the stakeholders’ expectations at local, regional, and global levels.

Literature (12–15) recognizes multiple types of OSH policy styles: (1) prescribed OSH policies and practices (2), outcome-based OSH policies and practices, and (3) systems-based OSH policies and practices.

Prescribed or prescriptive OSH policies mandate that business organizations and high-risk industries, such as energy and construction, strictly adhere to these prescriptive policies (12–15). For example, as listed in Table 1, Southeast Asia’s (Malaysia) Hazard Identification, Risk Assessment, and Risk Control (HIRARC)—a prescriptive OSH policy—and business organizations must adhere to these risk assessment guidelines in the region. Literature (12–15) notes that prescriptive policies may be more effective in high-risk industries.

**Table 1:** Comparison of regional OSH risk management policies, criteria, and specifications.

Regions items compared	North America			Southeast Asia		
	SK	CA	USA	MYS	SGP	THA
1. OSH laws and regulatory						
1.1 Enforcement agency	✓	✓	✓	✓	✓	✓
1.2 Main OHS laws	✓	✓	✓	✓	✓	✓
1.3 Regulation on OSH risk management	✓	✓	✓	–	✓	✓
1.4 Guideline/code of practices	✓	✓	✓	✓	✓	–
2. General requirements						
2.1 Application	✓	✓	✓	✓	✓	✓
2.2 Criteria for risk assessment team are specified	X	X	X	X	✓	✓
2.3 Risk assessment team needs to attend RA training	X	X	X	✓	✓	✓
2.4 RA training is given by regulatory certified training provider	X	X	X	X	✓	X
2.5 Roles of each key personnel are detailed out	✓	✓	✓	X	✓	X
3. Pre-assessment						
3.1 Classifying work activity	✓	✓	✓	✓	✓	✓
3.2 Work activity identification using prescribed form	X	X	X	X	✓	✓
4 Hazard identification						
4.1 Given examples of types of hazards	✓	✓	✓	✓	✓	✓
4.2 Given examples of hazard	✓	✓	✓	✓	✓	✓
4.3 Other factors for consideration in hazard identification	✓	✓	✓	X	✓	✓
5. Risk assessment						
5.1 Assessment approach	✓	✓	✓	✓	✓	✓
5.2 Severity description	✓	✓	✓	✓	✓	✓
5.3 Type of severity rubric	X	X	X	✓	✓	✓
5.4 Type of likelihood rubric	X	X	X	✓	✓	✓
5.5 Risk assessment matrix scale	X	X	X	✓	✓	✓
5.6 Levels of risk and risk matrix number (RMN)	X	X	X	✓	✓	✓
5.7 Prohibit activity classified as the highest risk level	✓	✓	✓	X	✓	✓
5.8 Workplaces are flexible to define their own likelihood and severity scale and matrix size	✓	✓	✓	✓	✓	X
6. Risk control						
6.1 Risk control method	X	X	X	✓	✓	✓
6.2 Reassessment of the highest risk level after additional controls	X	X	X	X	✓	✓
7. Communication and consultation						
7.1 Informing employees of the risk and results of assessment	✓	✓	✓	✓	✓	✓
7.2 Encourage workers’ participation and consultation	✓	✓	✓	✓	✓	✓
7.3 Encourage stakeholder participation and consultation	X	X	X	X	✓	✓

“✓” means present and “X” means no clear indication of the presence of the respective regional OSH risk management policies, criteria, and specifications. Adapted from references (42, 47, 53, 55, 57–63).

In contrast, North America’s OSH policies generally appear to be more outcome-based policies (13), also known as performance-based policies (12, 14). Outcome-based OSH policies are more flexible, offering industries and workplaces greater freedom to determine processes or procedures that meet or exceed regional expectations or goals for OSH policies and practices (12, 14, 15), systems-based OSH policies, such as ISO 45001: 2018-Occupational Health and Safety Management Systems, and ISO 31000: 2018-Risk management guidelines, are a couple of examples of systems-driven OSH policies and practices. Nevertheless, all (prescriptive, outcome-based, and systems-based) OSH policies have their pros and cons (12). Also, researchers are constantly exploring new approaches (16) in OSH policies that may be beneficial to high-risk industries.

This review analysis compares OSH policies in the six regions: three of the North American regions—Saskatchewan (SK), Canada (CA), and the United States of America (USA)—and three of the Southeast Asian regions—Malaysia (MYS), Singapore (SGP), and Thailand (THA). Saskatchewan is one of the provinces in Canada, whereas all other five countries are members of the International Labor Organization (ILO) (17). With the slogan of “Advancing social justice, promoting decent work” (18), the ILO promotes occupational safety and health (OSH) worldwide.

According to the ILO, work-related factors take the lives of about 2.93 illion workers every year, about 395 million workers suffer from a non-fatal workplace injury year after year, and 2.41 billion workers fight with extreme hot weather at workplaces (18) regularly. According to an older report, the ILO estimated approximately $3 trillion USD of annual damage due to workplace-related safety and health issues (19). In today’s context, the cost associated with OSH issues may be potentially significantly higher than $3 trillion USD per year.

The Workers’ Compensation Board (WCB) in Saskatchewan recently reported that the cost of workplace injury claims in 2024 alone was approximately $182.55 million USD (equivalent to $255 million CAD) (20). Unfortunately, the WCB-Saskatchewan failed to attain its target injury rate of 3.63 (20), p. 58 in that year. Canada recorded the national cost (cumulative cost of all provinces and territories in Canada) of approximately $7,089.30 million USD (equivalent to $9,904.1 million CAD) under the “benefits paid during the year” category in 2023 (21). Canada’s complete OSH cost-related data for 2024 was not available at the time of writing this report. According to the Damage Information Reporting Tool (DIRT), a committee within the Canadian Common Ground Alliance (CCGA), there were 39.8 facility damages on average per working day in 2023 (22), p. 6.

According to an estimate by the National Safety Council (NSC), the cost to the USA was approximately $176.5 billion (USD), which equates to $1,080 USD per worker and $1,460,000 USD per death (23, 24). According to the Bureau of Labor Statistics in the USA, as cited by the National Safety Council on its social media account, there were about 2.6 million workplace injuries in 2023 (23).

Likewise, the Southeast Asian regions also incur financial losses and suffer human suffering due to OSH incidents and accidents. For perspective, even though Malaysia’s most recent press release on OSH injuries and fatalities did not disclose or account for the financial burden caused by workplace related illness, injuries, and deaths (25), considering the approach of NSC above ($1,460,000 USD per death), approximately $473 million USD (324 fatalities × $1,460,000 per death) was the cost for workplace fatalities alone in 2023. Additionally, Malaysia experienced a significant increase (13.8% from the previous year) in workplace non-fatal injuries, totaling 38,626 in 2023 (25). Singapore lost $62.78 million USD (43 fatalities × $1,460,000 per death) in workplace fatalities, and there were also a total of 22,157 workplace injuries 22,157 (26). The authors were unable to collect Thailand’s current workplace OSH-related statistics and learned that the ILO also struggled to collect OSH statistics from Thailand (27). The last statistics reported by the ILO for Thailand were in 2020 and the ILO recorded 5.27 (~5.30) fatalities per 100,000 workers and a 761.59 non-fatal occupational injury rate per 100,000 in 2020 (28–30).

Furthermore, the current literature on OSH risk management is only confined to a comparison of main OSH laws among Southeast Asian countries, and there have been limited studies that extend beyond that (31, 32). There is a lack of or limited literature that has performed a comparative analysis of OSH policies between North American regions and Southeast Asian regions. In contrast, literature (33) advocates for a global “collaboration and alliance” (33), p. 372 to tackle OSH issues proactively and innovatively, and further research is required to understand an effective regulatory approach to enhance workplace health and safety performance (34).

Thus, there are three major purposes of this review study: (1) to compare and contrast OSH risk management policy and practice styles in the regions, (2) to explore opportunities in OSH risk management policies based on their recent OSH performance in the regions, and (3) to recommend a conceptual framework and or other opportunities to improve regional OSH policies and OSH performances continuously.

### 1.1 Definition of terminologies

- OSH policies: In this review study, OSH policies refer to the regional, national, or global occupational safety and health policies and risk management practices, acts, regulations, legislations, or laws.
- SDG indicator 8.1.1: SDG Indicator 8.1.1, also referred to as SDG 8.1.1, means the United Nations’ Sustainability Development Goals (SDGs) eighth goal, measured with workplace non-fatal and fatal injuries in each geographical region discussed in this study.
- ILO: ILO is the abbreviation of the Internal Labor Organization, an agency within the United Nations, that promotes the United Nations’ SDGs goals, such as SDG 8.1.1.
- OSH-MS: OSH-MS is the abbreviation of Occupation Safety and Health Management System, which is a proactive system or framework that supports workplaces to attain their safety and health related both short term (e.g., it guides workers on how to perform day to day worksite hazard assessment and control) and long term (e.g., it structurally guides workplaces on how to achieve overall improved safety and health performances).
- HIRARC: HIRARC is the abbreviation of Hazard Identification, Risk Assessment, and Risk Control as defined by Malaysia’s governmental guidelines for workplace hazard assessment and control policies in 2008.
- Damage prevention: In this study, damage prevention means a proactive approach or initiative to prevent or minimize workplace incidents, accidents, human suffering, including damage to the workplace equipment and infrastructure.

## 2 Methods, data collection, and data analysis

In this systematic mixed method comparative review study, the authors utilized Preferred Reporting Items for Systematic Review and Meta-Analyses (PRISMA) (35), a credible tool in academic research (36, 37), to search, screen, retrieve, and include the six regional OSH policies to compare as well as evaluate the effectiveness of the OSH policies in the regions. The authors minimized the search bias by developing search questions and literature’s eligibility criteria (both inclusion and exclusion) based on the Population, Intervention, Comparison, Outcome, and Study (PICOS) framework (38).

**Figure 1:**
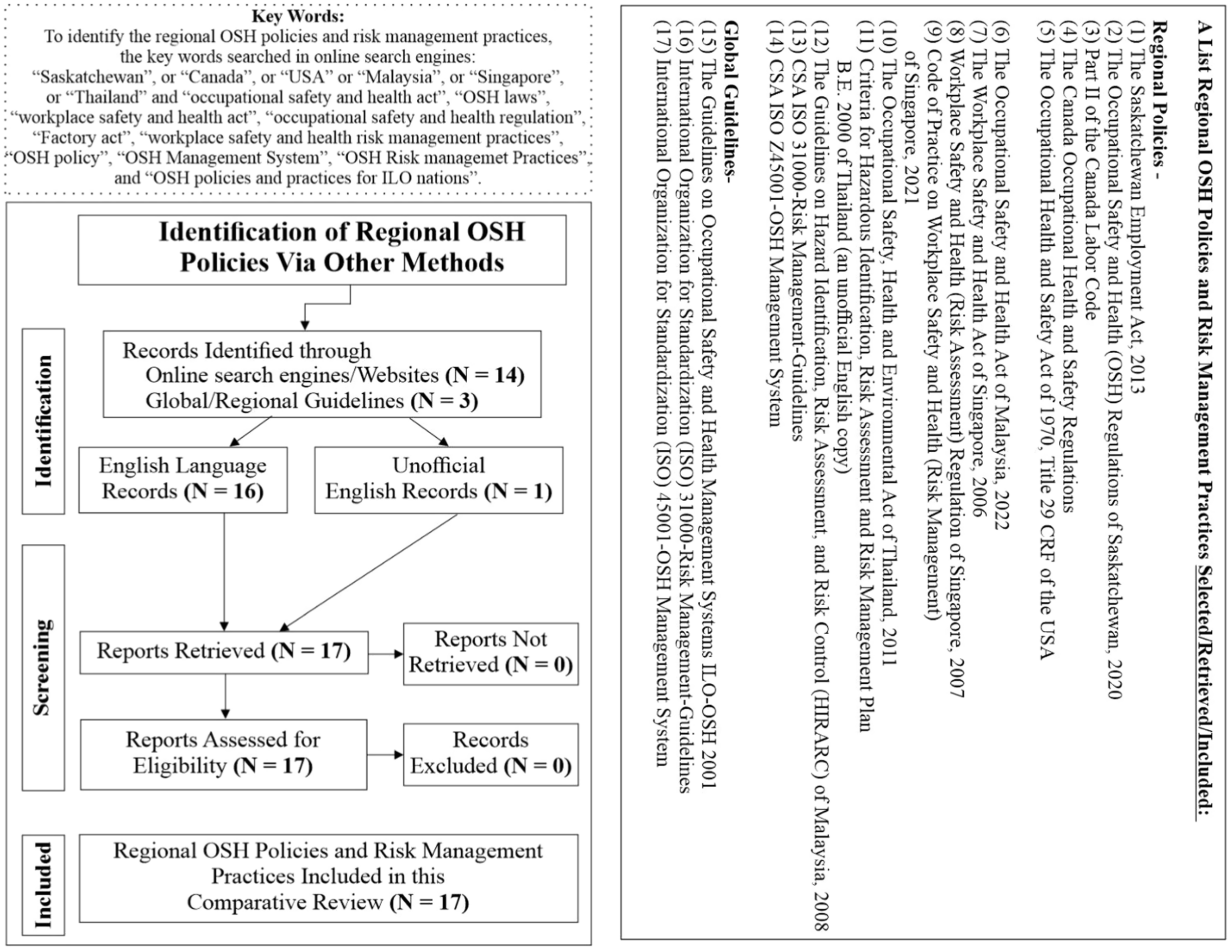
PRISMA flow diagram. Adapted from references (35–37).

In this review, Population (P) referred to the employed persons or workers, including OSH Inspectors in Noth America (Saskatchewan, Canada, and the USA) and the Southeast Asia (Malaysia, Singapore and Thailand), Intervention (I) referred to the regional OSH policies and risk management policies and practices, Comparator (C) represented the specific types or styles, such as prescribed and performance based regional OSH policies, Outcome (O) referred to the SDG 8.1.1—Non-fatal occupational injuries per 100,000 workers, and SDG 8.1.1—Fatal occupational injuries per 100,000 Workers, and Study (S) represented a high-level mixed method comparative review of regional OSH policies. This review excluded the literature search in standardized databases, such as Scopus and Web of Science because eligible documents or literature in this review study were current regional or national OSH policies written in the English language. Therefore, the authors primarily utilized online search engines to search such documents and the respective regions’ official websites to verify the authenticity of the English do. Thus, the authors excluded any non-English and non-official regional OSH policy and risk management practices documents in this review. See PRISMA diagram for details (Figure 1).

Identification: To identify the regional OSH policies, the authors used online search-engines with keywords (individually and or multiple phrases like Boolean search method): “Saskatchewan” “Canada,” “USA,” “Malaysia,” “Singapore,” “Thailand,” “occupational safety and health act,” “OSH laws,” “workplace safety and health act,” “occupational safety and health regulation,” “factory act,” “workplace safety and health risk management practices,” “OSH risk management guidelines,” “OSH policy,” “OSH management system,” “OSH risk management practices,” or “OSH policies and practices for ILO nations.” Additionally, the authors conveniently selected ISO 31000-Risk Management Guidelines, ISO 45001 OSH Management System, and the Guidelines on Occupational Safety and Health Management Systems ILOOSH 2001, for their global as well as regional recognition in workplace safety and health. Additionally, the authors searched and selected Malaysia’s Guidelines for HIRARC 2008 and Thailand’s Criteria for Hazardous Identification, Risk Assessment, and Risk Management Plan B.E. 2000 an unofficial version of English translated copy by Japan External Trade Organization (JETRO), due to their relevance in this comparative study.

Screening: The authors screened only English, including one non-official English document. Verified screened documents were the regional OSH policies associated with risk management practices in the respective regions’ official publicly available webpages, and thus, selected only regional OSH policy documents for this comparative analysis.

Included: The authors included a total of 17 OSH policy documents relevant to the comparison of the six regional OSH policies in this study.

### 2.1 Data collection and measurement

While synthesizing the qualitative data associated with OSH policies of six regions, the authors used nine themes: (1) OSH risk management regulatory framework, (2) the scope and general requirements, (3) pre-assessment evaluation, (4) hazard identification, (5) risk assessment methodology, (6) risk control, (7) communication and consultation, (8) documentation and record keeping, and (9) reviewing the risk assessment for qualitative comparative analysis of regional OSH policies. Table 1 reflected the qualitative assessment of the policies.

Regarding the quantitative analysis of the effectiveness of OSH policies in the regions, the authors measured the OSH performances of the OSH policies in the respective areas with three quantitative data retrieved from 2009 to 2025 from ILO’s online webpage: (1) SDG 8.1.1—Non-fatal occupational injuries per 100,000 Workers, (2) SDG 8.1.1—Fatal occupational injuries per 100,000 Workers, and (3) OSH Inspectors per 10,000 Employed Persons. The authors requested and obtained Saskatchewan’s OSH performance data from the Ministry of Labor Relations and Workplace Safety, OSH Division in Saskatchewan on May 14, 2025.

### 2.2 Data analysis

The authors used IBM SPSS version 30.0 software for quantitative general multivariate regression analysis. IBM SPSS software is a credible data analysis tool commonly used in regression analysis studies to compute descriptive statistics, test hypotheses, and to perform the test hypotheses, and to perform regression analysis to determine the causal relationship between the OSH inspectors to employed person ratios (IV) and SDG 8.1.1 (DV).

For data triangulation or validation and to minimize the individual biases in this review report, multiple authors independently analyzed, reviewed, and approved of this report’s entirety. Additionally, the authors sought and received expert feedback from the Universiti Putra Malaysia’s (UPM) Faculty of Medicine and Health Sciences, Department of Chemical and Environmental Engineering, Faculty of Engineering, and the Occupational Safety and Health Division of the Ministry of Labor Relations and Workplace Safety, Government of Saskatchewan, Canada.

## 3 Results and discussion

Six geographical regions, namely, Saskatchewan, Canada, the USA, Malaysia, Singapore, and Thailand, shared the commitment of creating safer and healthier workplaces for all stakeholders, even though they differed on OSH policy styles, concepts, and ways to execute the OSH policies to generate the OSH values in the regions. This section presents a brief overview of both qualitative and quantitative results of the research, discussing the findings, which begin with an examination of OSH-MS and Risk Management Practices in the regions.

### 3.1 OSH-MS and risk management practices

OSH-MS provides overall systematic frameworks to guide business organizations to achieve their OSH compliance or performance (39–41). ILO’s OSH-MS framework and International Organization for Standardization’s (ISO) ISO 45001 offer guidelines to prevent or minimize OSH risks, whereas ISO’s 31,000 provides a sub-system called the risk assessment process for OSH-MS.

Implementing OSH risk management is perceived as enabling employers to manage risk in a structured way, preventing or reducing occupational accidents and diseases, promoting workplace OSH culture, and improving workplace productivity and performance (42). Risk assessment is also known as the process of evaluating occupational safety and health risks associated with a work activity’s hazards and determining the appropriate measures to prevent or control the risk (43). According to the ISO, risk management involves five main processes, as defined in ISO 31000. Additionally, ISO 31010 specifies 38 techniques involving risk assessment in detail (10, 44).

Southeast Asia’s Malaysia, Thailand, and Singapore adhere to the ILO’s guidelines on OSH-MS and national guidelines to customize the workplace-specific OSH-MS for the organizations. As part of the strategy to improve occupational safety and health conditions at the workplace and prevent damage, the ILO recommended the need for workplace risk management among member nations. In this regard, the ILO published Guidelines on OSH-MS in 2001 (45) and thereafter developed A *5-Steps Guide* (46) on conducting workplace risk assessments in 2014. Both guidelines serve as a reference for governments to adapt the framework on OSH Risk Management into their national OSH policy (44, 47).

#### 3.1.1 North American OSH-MS

Saskatchewan, Canada, and the USA provide minimum or general OSH programs or set expectations that workplaces must satisfy through respective OSH policies. However, high-risk organizations in the oil and gas industry and construction in Canada, as well as those in the province of Saskatchewan, have options to follow OSH-MS and risk management practices customized by industry safety associations and/or the Canadian version of ISO 45001:2018 and ISO 31000:2019. ISO 31000 and 45001 are international standards, and high-risk industries in the USA have options to incorporate these international standards to realize higher workplace safety and health objectives. OSH policies in Canada, as outlined in the Saskatchewan Employment Act 2013, OSH Regulations 2020 (SK), and the Labor Canada Code II, and in the USA, through OSHA, provide generic OSH safety and health programs.

#### 3.1.2 Risk management practices

North American regions have different approaches to OSH risk management practices compared to Malaysia, Singapore, and Thailand. For example, high-risk industries in Canada and the USA have the flexibility to incorporate ISO 31000 or risk management practices customized or recommended by regional safety associations or industries, or customized pre- and post-job hazard analyses by workplaces. Whereas the Southeast nations generally adopt semi-quantitative methods such as HIRARC, regional OSH policy expectations are adhered to the ILO’s guidelines and national policies for risk management practices. See Table 1 for details.

Based on the qualitative findings listed in Table 1, the authors posited that the OSH policies in North American regions were outcome-based, whereas more prescriptive OSH policies dominated the Southeast Asian regions and workplaces.

### 3.2 Qualitative findings

#### 3.2.1 North America—Saskatchewan and Canada

The province of Saskatchewan has its own stand-alone OSH policies, outlined in the Employment Act and OHS Regulations, 2020. The OHS Regulations 2020 provide guidelines on various types of work, expectations for worker training, and protective equipment for workers in all types of workplaces, including high-risk industries, such as the oil and gas industry. Saskatchewan’s OSH Division, under the Ministry of Labor Relations and Workplace Safety, is the enforcement or regulatory body. Whereas, at the federal level, the Department of Employment and Social Development (ESDC) enforces Part II of the Canada Labor Code and the Canada Occupational Health and Safety Regulations for workplace health and safety issues. Canadian OSH policies (acts and regulations) to some extent set guidelines for best practices and expectations pertaining to certain workplace activities, such as entering confined spaces in the oil and gas industry (Part 18, Confined Space, Section 18 of OSH Regulations 2020, Saskatchewan). However, industries, safety associations, workplaces, and the CSA Group may develop and disseminate the risk management practices and guidelines, such as CSA ISO 31000:18-Risk Management Guidelines and CSA ISO Z45001:19-OSH Management System.

The federal and provincial OSH policies and practices apply to all provincial and federal workplaces, except the Canadian military forces. In terms of criteria for risk assessment teams, both provincial and federal policies do not specify if risks must be assessed by a team and the structure of such teams, though Canadian OSH policies clearly extend workers the right to participate in OSH activities, and in return, industries and workplaces encourage workers to participate actively in OSH activities, such as pre-job planning and risk assessments with other workers at the jobsites. Additionally, the Canadian OSH policies mandate employers to ensure their workers are trained or competent or have direct supervision for the assigned tasks, and trained OHC members may perform workplace risk assessments. Generally, private safety and health training centers, higher education institutions (colleges, universities, etc.), and health and safety associations offer such risk assessment training. Industries and workers can receive risk assessment (RA) training from WorkSafe Saskatchewan’s safety training and the Canadian Centre for Occupational Health and Safety (CCOHS).

There is no direct reference to WorkSafe Saskatchewan and CCOSH in the OSH policies. Canadian policies outline the regulatory roles and responsibilities of key personnel, such as employers, owners, supervisors, workers, prime contractors, operators, and suppliers, and such policies also classify work activities, such as working at heights, construction, rigging, blasting, tunneling, and drilling. However, the OSH policies do not provide industries and workers with work activity identification forms, and policies expect companies and industries to practice such internally developed forms. Some industries and workplaces provide pre-developed checklists with examples, and others may practice blank forms that workers can freely document in their workplaces or task-specific types of hazards. Workers are encouraged to identify all potential risks at the workplace, but the terrorist threats are not generally considered in hazard identification.

The OSH policies in Canada do not specify an assessment approach, types of severity, likelihood rubrics, risk-assessment matrix scales, levels of risk, risk matrix numbers, risk control methods, or reassessment of the highest risk level after implementing additional controls. Industries and workplaces employ a semi-quantitative and qualitative hazard assessment approach, including the assessment of multiple severity descriptions, such as bodily injuries, health-related illness, potential environmental impact, and equipment and property damage. However, industries, workplaces, and workers generally determine all those factors: risk assessment approach, severity, likelihood rubrics, risk assessment matrix scales, levels of risk, and risk matrix numbers. Workers also have the right to refuse jobs that are unusually dangerous in Canada. High-risk workplaces and their workers also determine risk control methods using the five-step hierarchy of control. Industries and workplaces have the monopoly to determine how long they should keep such records for and how often such documents should be reviewed. Workplaces prohibit workers from performing hazardous tasks without safely mitigating the risks. In the case of high-risk, dangerous work refused by workers, the OSH policies expect the workplaces to inform the other workers prior to assigning the tasks of why the dangerous tasks were refused to be performed.

Again, Canadian OSH policies consider workplace health and safety issues and risk management practices to be shared responsibilities and encourage workers’ participation and consultation with the health and safety committees as required, if/when required. Although the policies do not specify the participation of other stakeholders, such as the public, industries, and workplaces, they generally encourage all stakeholders to make the workplaces safer, healthier, compliant with OSH policies, and more damage prevention-proof.

#### 3.2.2 North America—USA

Like Canada, the USA has its federal Occupational Health and Safety Act of 1970, which was recently amended in 2021. Title 29 CRF 1910–OSH General Industry, 29 CFR 1926–OSH Construction, and 29 CFR 1928–OSH Agriculture-related regulations in the USA. Such OSH policies are applicable at all workplaces except in states, local government agencies, and the US army/military. The U.S. Department of Labor enforces the OSH policies and laws. Likewise in Canada or Saskatchewan, the USA has quite similar OSH risk assessment guidelines or practices, criteria for risk assessment teams, risk assessment team’s training or qualifications, who can or cannot offer risk assessment training, legislative roles or responsibilities of key personnel in the OSH policies, pre-risk assessment, hazard identification approaches, risk assessment practices, risk control strategies, promotion of communication and consultation regarding the OSH risk management practices, requirements or expectations on documentation and record-keeping, and revision of such OSH risk management practices. In short, just like in Canada, OSHA in the USA sets minimum workplace safety standards, and it is the industry or business organizations that determine the type of OSH risk management frameworks and practices, such as ISO 31000 and ISO 45001, that are more effective for their industries or workplaces.

#### 3.2.3 Southeast Asia—Malaysia

OSH in Malaysia is enforced by the Department of Occupational Safety and Health (DOSH) under the Ministry of Human Resource and the main OSH legislation is the Occupational Safety and Health (Amendment) Act 2022 (48). As part of the effort to reduce occupational accidents, DOSH introduced guidelines on Hazard Identification, Risk Assessment, and Risk Control (HIRARC) in 2008 to encourage business organizations to implement risk management, especially small and medium enterprises (SME) (49). Though adapting Guidelines for HIRARC 2008 for risk management is not mandatory, DOSH encourages all business sectors to implement HIRARC. The prescriptive guidelines for HIRARC 2008 utilizes a semi-quantitative risk assessment method, and the process of risk management involves four main stages: firstly, classifying work activities, followed by identifying hazards, then risk assessment, and finally, implementation of control measures when necessary (50, 51). Prior to performing HIRARC, the guidelines emphasize employers assigning one trained personnel to lead the assessment.

After determining the work activities, under this guideline, the workplaces identify hazards. There are several hazard identification techniques as suggested in the guideline, such as job hazard analysis, failure analysis, and potential accident factors. The organizations also have the flexibility to use other types of hazard identification techniques according to their own preference. The guideline highlights three main hazards, namely safety, health, and environmental hazards. In risk analysis, the guideline recommends using a five-times-five risk matrix, and offers severity as well as likelihood description in a holistic rubric form with a scale of 1–5. The severity description only indicates injury and property damage. There are three levels of risk: low, medium, and high. Risks that are classified as high require immediate attention and action to control the hazard. Guidelines for HIRARC 2008 adopt the hierarchy of control method in suggesting measures to eliminate the hazard or reduce the impact of the hazard. Workplaces require documenting the process of risk assessment using the prescribed form and maintaining the documents accordingly. Workplaces require to review HIRARC periodically, at least every 3 years. Workplaces must communicate the result of the risk assessment with the workers who are affected by the hazard, and monitor the implementation of control measures (42, 51, 52).

#### 3.2.4 Southeast Asia—Singapore

The main occupational safety and health law in Singapore is the Work Safety and Health (WSH) Act 2006, and the Occupational Safety and Health Division (OSHD) under the Ministry of Manpower enforces this law. To address the need for OSH risk management, Singapore enacted the Work Safety and Health (Risk Assessment) Regulation in 2007. This regulation is applicable to all workplaces that are covered by the WSH Act (32). According to this regulation, workplaces need to conduct risk assessments before initiating any new work, regardless of whether it is routine or non-routine. The Code of Practice on Workplace Safety and Health (WSH) (Risk Management) briefly illustrates the methodology to comply with the WSH (Risk Assessment) Regulation. Singapore has revised the Code of Practice on WSH 3 times since 2011, and it sets the minimum requirement for workplace risk management in Singapore (53). Code of Practice in WSH (Risk Management) also adopts a semi-quantitative methodology. A risk management team assigned by the employers shall conduct risk assessment in Singapore, and the team shall consist of personnel from multidisciplinary areas of work.

Furthermore, the team leader needs to complete the regulatory-approved risk management course from a registered training provider. In accordance with the Code of Practice, the first step is to identify all work activities in the organization, and it needs to be put on record in the inventory of work activities form. The risk management team may include systematic process reviews, Process Hazard Analysis (PHA), Job Observations, and Job Safety Analysis (JSA) while performing hazard identification as recommended in the Code of Practice. Additionally, workplaces shall document the risk assessment using the risk assessment form as indicated in the Code of Practice. In risk analysis, the Code of Practice applies the common five-times-five-risk assessment matrix in determining the level of severity and likelihood, and the severity and likelihood are in a holistic rubric table with a 1–5 scale.

Furthermore, the classification of each severity level in Singapore’s Code of Practice is inclusive of occupational health-related criteria. Hence, this will give the industry clarity and precise selection in determining the risk level of both injuries and illnesses. Singapore’s risk assessment Code of Practice is also much more stringent and conservative, whereby work activities that are classified as high risk are not allowed to start unless the risk level has been reduced to a lower level. In the Code of Practice, the selection of control measures is based on the hierarchy of control. Organizations are required to review their risk assessment every 3 years. In the event that there is any workplace injury or significant changes to work practice or procedures, the workplace needs to review the risk assessment earlier. The workplace requires to inform the workers risk assessment results, including the risks associated with the tasks and control measures in place (45).

#### 3.2.5 Southeast Asia—Thailand

There are three main ministries that are involved in occupational safety and health-related issues in Thailand. They are the Department of Labor Protection and Welfare, Social Security Office, and Occupational Safety and Health Committee that sit under the Ministry of Labor, the Bureau of Occupational and Environmental Disease that falls under the purview of the Ministry of Public Health, and the Department of Industrial Works, Office of the Permanent Secretariat, and Industrial Estates Authority that sit under the Ministry of Industry (54).

The Department of Industrial Work Rules on Criteria for Hazardous Identification, Risk Assessment, and Risk Management Plan B.E. 2,543, 2000 (55) regulates the occupational risk management in Thailand. However, this regulation is only applicable to factories that are under the purview of the Department of Industrial Works. The regulation specifies that factory employers are required to appoint at least three workers to conduct the risk assessment, who possess qualifications as prescribed in the regulation. The regulation does not restrict the types of hazard identification methods. Nevertheless, it has comprehensively recommended several methods, such as the Checklist method, What-If analysis, Hazard and Operability Study (HAZOP), Fault Tree Event Analysis (FTEA), Failure Mode Event Analysis (FMEA) and Event Tree Analysis as depicted in regulation. This regulation intends to incorporate a semiquantitative approach, which applies a four-time-four risk assessment matrix based on the severity and probability level of the event. There are four holistic rubric scales for probability level that include the frequency of each occurrence.

In contrast, the impact of severity is classified into four individual events based on whether the event is affecting the people, community, environment, or property. There are four risk levels, beginning with the lowest risk, known as small risk, acceptable risk, high risk, and finally, unacceptable risk (56). Work activity that is categorized as unacceptable risk will need to cease operation, and the workplace immediately implements the corrective actions to reduce the risk. Subsequently, the regulation also requires employers to consistently monitor the implementation of risk management plans for work activities classified as high risk. The risk management plan mentioned in the regulation refers to the risk reduction plan and risk control plan to which the factory employers shall apply so that the risk of the identified hazard can be reduced and controlled.

The risk control in this regulation is based on principles of prevention, with nine procedural steps for prevention and control measures. The workplace shall document the risk assessment using prescribed forms in the regulation and review the risk management plan every 5 years, once the factory applies for a renewal of the factory license. However, the regulation does not consistently mention the need for a review when an accident happens.

### 3.3 Quantitative analysis – regional OSH injury rates

This research initially made an effort to collect the regional OSH performance data, such as trending of total injury rates (TIRs), total lost time injury rates (TLTIR) and the Ratio or Rate of OSH Inspectors (ROI) per 10,000 in North America and the Southeast Asia for the duration of 2020 to 2025, to evaluate the effectiveness of OSH policies in the regions. However, this review study observed nonuniformity in workplace OSH-related publicly available data between the regional agencies, and not all regions, except Saskatchewan, had publicly available data consistently from 2020 to 2024, while writing this report. Canada’s OSH-related data, such as total injury rates, were not publicly available for 2023 and 2024 while writing this report.

Then, this research retrieved dataset from ILO’s publicly available datasets and created Table 2– OSH Risk Management Policy Performance – Quantitative Comparison. The authors also requested Saskatchewan’s similar OSH performance data and obtained the dataset from the Ministry of Labor Relations and Workplace Safety, OSH Division in Regina, Saskatchewan on May 14, 2025. Thus, the authors reported the quantitative comparative analysis of the effectiveness of the regional OSH policies based on the three OSH performance data: SDG 8.1.1—non-fatal injuries per 100,000 workers; SDG 8.1.1—fatal injuries per 100,000 workers; and inspectors per 10,000 employed persons, publicly available through the ILO’s online website.

**Table 2:** OSH risk management policy performance—quantitative comparison.

Regions		Non-fatal injuries per 100,000 workers	Fatal injuries per 100,000 workers	Inspectors per 10,000 employed persons
North America	SK	3,905.3	6.09	1.13
	CA	1,463.50	5.0	0.1
	USA	1,805.0	3.7	0.1
Southeast Asia	MYS	3,229.7	14.6	0.5
	SGP	622.0	1.0	1.2
	THA	761.6	5.3	0.2

Adopted from references (64, 65) and the data were received from the Occupational Safety and Health Division, Ministry of Labor Relations and Workplace Safety, Government of Saskatchewan (SK), Canada.

**Figure 2:**
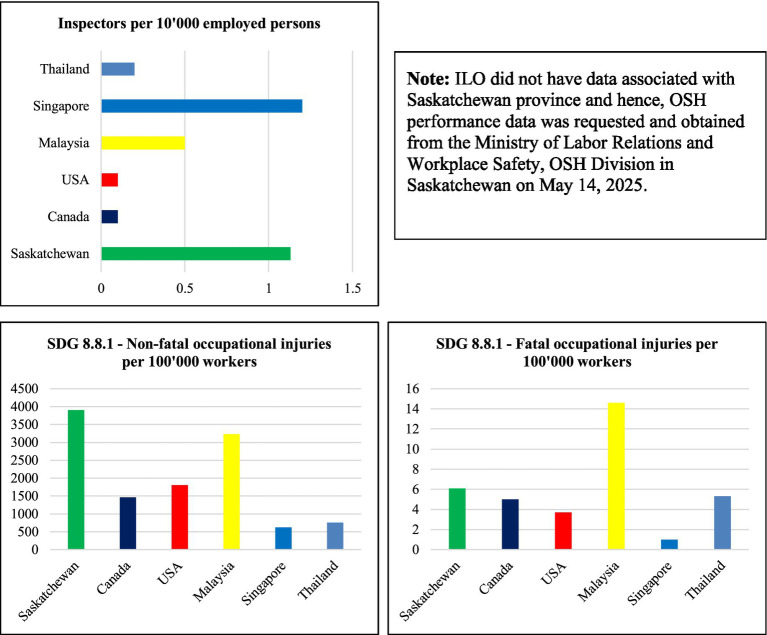
OSH risk management policy performance—graphical representation. Figure was developed to provide visual representation of data associated with Table 2. ILO did not have data associated with Saskatchewan province and hence, OSH performance data was requested and obtained from the Ministry of Labor Relations and Workplace Safety, OSH Division in Saskatchewan on May 14, 2025.

Despite the apparent limitations or weaknesses associated with the secondary self-reported regional data, the authors posited that Table 2-OSH Risk Management Policy Performance—Quantitative Comparison offered, at minimum, a snapshot of the effectiveness of the regional OSH policies. See Table 2 and Figure 2 for details.

As illustrated in Table 2 and represented in Figure 2, Singapore, an industrialized member nation of ILO, which largely incorporates prescriptive OSH policies had the lowest non-fatal injury rate (622 per 100,000 Workers), the lowest fatal injury rate (1 per 100,000 Workers), and highest ratio of OSH inspectors (1.2 inspectors per 10,000 employed persons) between all six regions compared in this review analysis.

In the non-fatal injury rate per 100,000 workers category, the other lowest ranking regions: Thailand (761.6), Canada (1,463.50), the USA (1,805.0), Malaysia (3,229.7), and Saskatchewan (3,905.3) ranked second, third, fourth, fifth, and sixth, respectively. Whereas in the fatal injury rate per 100,000 Workers category, USA (3.7), Canada (5.0), Thailand (5.3), Saskatchewan (6.09), and Malaysia (14.6) scored second, third, fourth, fifth, and sixth lowest ranks, respectively. Saskatchewan (1.13), Malaysia (0.5), Thailand (0.2), Canada (0.1), and the USA (0.1) ranked second, third, and fourth (Canada and the USA had the same ranking with equal ratio in this category), respectively, in the OSH inspectors per 10,000 employed persons category.

Thus, the quantitative OSH performance indicator analysis performed, so far, in this review validated the existing literature, such as the findings in Barua and Mannan (12). As in Barua and Mannan (12), this review analysis was unable to determine which styles of OSH policies (more prescriptive, or more performance-based) could generate higher policy compliance and attain shared OSH objectives of all stakeholders, including the regional government agencies and business organizations.

Singapore has the lowest injury rates among six regions and the highest ratios of OSH inspectors to employed workers in the regions further raising the question of whether there is a significant relationship between the number of OSH Inspectors and the regional occupational fatality and non-fatal injuries. Additionally, Singapore was one of the few countries or regions that constantly monitored the progress and opportunities against other higher performing countries internationally, such as countries members with the Organization for Economic Co-operation and Development (OECD). Singapore’s 3-year average workplace fatality rates are higher than the Netherlands, United Kingdom, Sweden, and Germany, but Singapore outperformed Australia, Japan, New Zealand, and the Republic of Korea (26), p. 7. Singapore not only records internally but also reports publicly the total number of dangerous occurrences (an OSH leading indicator) and businesses that are closely monitored by the ministry due to their workplace OSH performance. For example, Singapore had 19 dangerous occurrences and closely monitored eight workplaces because of OSH performance (26), p. 12. Singapore’s consistent proactive actions and greater transparency in OSH statistics might differentiate it from other regions.

Then again, not all Southeast regions equally enjoyed comparatively lower injury rates, and not all Southeast regions had higher ratios of OSH Inspectors to Workers than North American regions. For example, Saskatchewan had the second-highest OSH Inspectors to employed person ratio (1.13 per 10,000 Employed Persons), yet Saskatchewan had the highest non-fatal workplace injury rates and second-highest fatal injury rates among the six regions.

Therefore, the authors briefly made a scholarly attempt to investigate the relationship between the regional OSH Inspectors, occupational fatal injury rates, and non-fatal injury rates with a quantitative regression analysis.

#### 3.3.1 Singapore’s quantitative regression analysis

**Figure 3:**
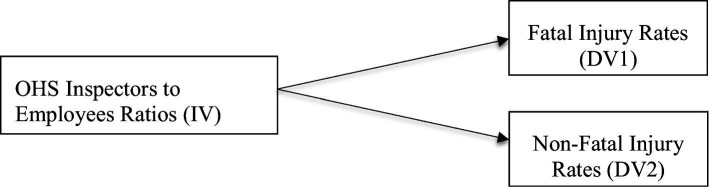
General multivariate regression analysis of research framework (OSH inspectors as predictor of regional SDG 8.1.1). ILO’s statistics showed that SDG 8.1.1 had two categories: fatal injury rates and non-fatal injury rates.

For this, the additional regression analysis in this report, the conceptual framework, major research question, and hypothesis are provided as follows. For details, see Figure 3.

Research question: How significantly do the ratios of OSH inspectors to employed workers (independent variable) predict the occupational fatal injury rates (DV1) and non-fatal injury rates (DV2) in the regions?

Hypothesis: The ratios of regional OSH inspectors to employed workers (independent variable) can significantly predict the occupational fatal injury rates (DV1) and non-fatal injury rates (DV2).

Null hypothesis: There is no significant relationship between the number of OSH inspectors (independent variable) and the occupational fatal injury rates (DV1) and non-fatal injury rates (DV2) in the regions.

The annual datasets SDG 8.1.1 retrieved on May 19, 2025, from ILO’s websites for the period of 2014 to 2025 associated with Singapore were more credible and complete datasets than the datasets related to other regions: Canada, the USA, Malaysia, and Thailand. Therefore, the authors tested the hypothesis and performed general linear model (GLM) regression analysis or general multivariate regression using IBM SPSS version 30.0 software on ILO’s statistics related to Singapore.

**Figure 4:**
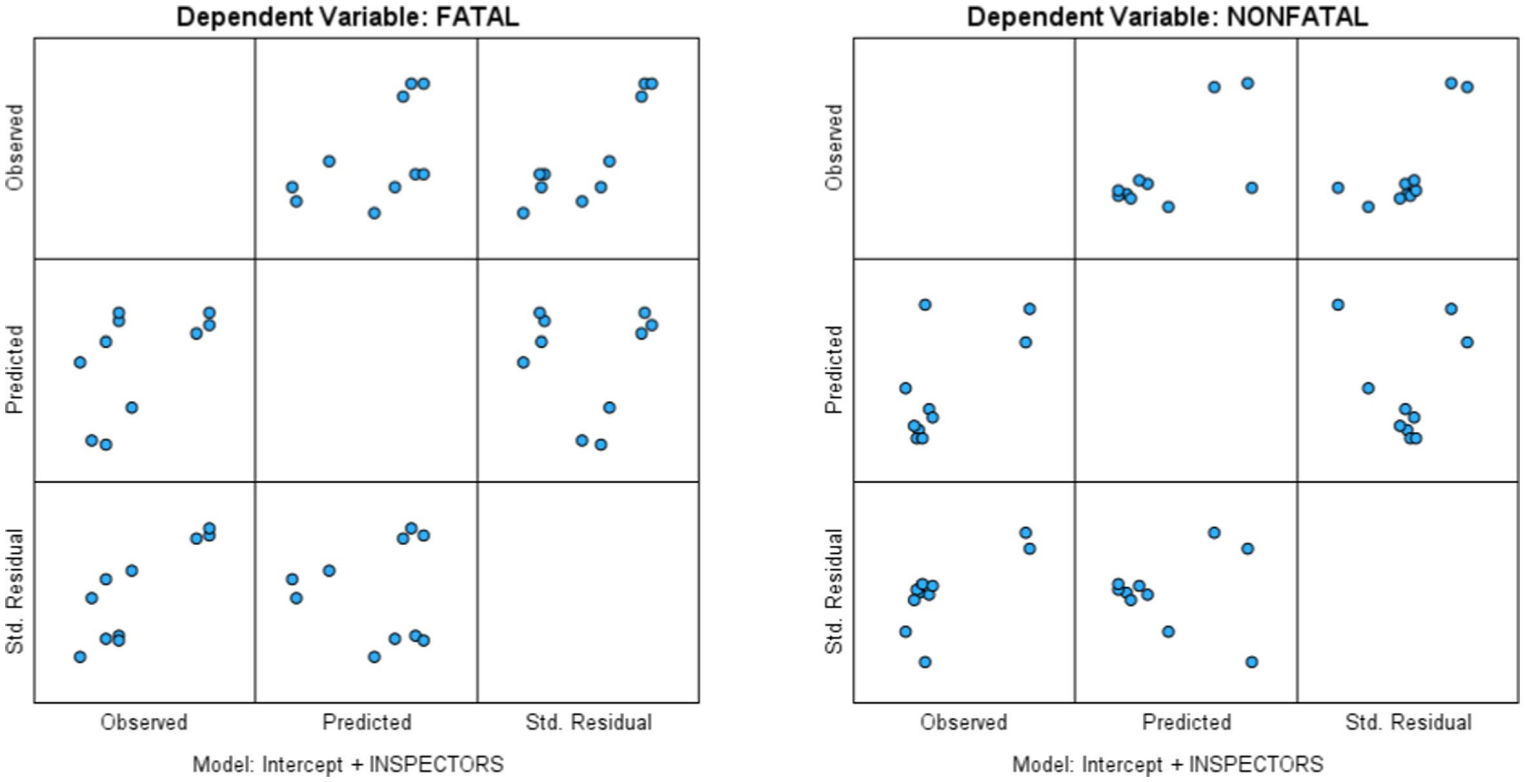
Observed × Predicted × Standard. Residual plots.

Table 3 represents the descriptive statistics associated with the variables: OSH Inspectors to Employed Persons per 10,000 ratios (IV), and SDG 8.1.1 [fatal injury rates (DV1), and non-fatal injury rates (DV2)]. See Table 3 for details. Additionally, see Table 4 for the test of normality, Table 5 for multivariate tests, Table 6 for tests of between-subjects effects, Table 7 for parameter estimates, and Figure 4 for observed predicted standard residual plots for details.

**Table 3:** Descriptive statistics—Singapore’s OSH inspector to workers ratios and SDG 8.1.1.

Variables			Statistic	Standard error
OSH inspectors	Mean		0.9950	0.03976
	95% Confidence interval for mean	Lower bound	0.9051	
		Upper bound	1.0849	
	5% Trimmed mean		0.9900	
	Median		0.9400	
	Variance		0.016	
	Standard deviation		0.12572	
	Minimum		0.88	
	Maximum		1.20	
	Range		0.32	
	Interquartile range		0.24	
	Skewness		0.886	0.687
	Kurtosis		−0.939	1.334
Fatal injury rates	Mean		1.3390	0.12069
	95% Confidence interval for mean	Lower bound	1.0660	
		Upper bound	1.6120	
	5% Trimmed mean		1.3322	
	Median		1.2000	
	Variance		0.146	
	Standard deviation		0.38165	
	Minimum		0.90	
	Maximum		1.90	
	Range		1.00	
	Interquartile range		0.75	
	Skewness		0.728	0.687
	Kurtosis		−1.227	1.334
Non-fatal injury rates	Mean		424.4000	32.63100
	95% Confidence interval for mean	Lower bound	350.5835	
		Upper bound	498.2165	
	5% Trimmed mean		417.9444	
	Median		383.0000	
	Variance		10,647.822	
	Standard deviation		103.18829	
	Minimum		343.00	
	Maximum		622.00	
	Range		279.00	
	Interquartile range		89.00	
	Skewness		1.661	0.687
	Kurtosis		1.192	1.334

In Tables 3–7, OSH inspectors represent the regional OSH inspectors (or labor inspectors) per 10,000 employed persons, fatal injury rates represent the fatal injuries per 100,000 workers, and non-fatal injuries represent the non-fatal injuries per 100,000 workers.

**Table 4:** Test of normality.

Variables	Kolmogorov–Smirnov test^a^			Shapiro–Wilk test		
	Statistic	df	Significance	Statistic	df	Significance
OSH inspectors	0.240	10	0.108	0.824	10	0.029
Fatal injury rates	0.242	10	0.099	0.837	10	0.041
Non-fatal injury rates	0.382	10	<0.001	0.669	10	<0.001

^a^Lilliefors significance correction.

**Table 5:** Multivariate tests^a^.

Effect		Value	*F*	Hypothesis df	Error df	Significance
Intercept	Pillai’s trace	0.588	4.986^b^	2.000	7.000	0.045
	Wilks’ lambda	0.412	4.986^b^	2.000	7.000	0.045
	Hotelling’s trace	1.425	4.986^b^	2.000	7.000	0.045
	Roy’s largest root	1.425	4.986^b^	2.000	7.000	0.045
OSH Inspectors	Pillai’s trace	0.579	4.819^b^	2.000	7.000	0.048
	Wilks’ lambda	0.421	4.819^b^	2.000	7.000	0.048
	Hotelling’s trace	1.377	4.819^b^	2.000	7.000	0.048
	Roy’s largest root	1.377	4.819^b^	2.000	7.000	0.048

Effect		Partial eta squared	Non-centrality parameter	Observed power^c^
Intercept	Pillai’s trace	0.588	9.972	0.613
	Wilks’ lambda	0.588	9.972	0.613
	Hotelling’s trace	0.588	9.972	0.613
	Roy’s largest root	0.588	9.972	0.613
OSH inspectors	Pillai’s trace	0.579	9.638	0.597
	Wilks’ lambda	0.579	9.638	0.597
	Hotelling’s trace	0.579	9.638	0.597
	Roy’s largest root	0.579	9.638	0.597

^a^Design: Intercept + Inspectors. ^b^Exact statistics. ^c^Computed using alpha = 0.05.

**Table 6:** Tests of between-subjects effects.

Source	Dependent variable	Type III sum of squares	df	Mean square	*F*	Significance
Corrected model	Fatal injury rates	0.359^a^	1	0.359	3.016	0.121
	Non-fatal injury rates	41,172.871^b^	1	41,172.871	6.026	0.040
Intercept	Fatal injury rates	1.207	1	1.207	10.145	0.013
	Non-fatal injury rates	1,742.306	1	1,742.306	0.255	0.627
OSH inspectors	Fatal injury rates	0.359	1	0.359	3.016	0.121
	Non-fatal injury rates	41,172.871	1	41,172.871	6.026	0.040
Error	Fatal injury rates	0.952	8	0.119		
	Non-fatal injury rates	54,657.529	8	6,832.191		
Total	Fatal injury rates	19.240	10			
	Non-fatal injury rates	1,896,984.000	10			
Corrected total	Fatal injury rates	1.311	9			
	Non-fatal injury rates	95,830.400	9			

Source	Dependent variable	Partial eta squared	Non-centrality parameter	Observed power^c^
Corrected model	Fatal injury rates	0.274	3.016	0.334
	Non-fatal injury rates	0.430	6.026	0.578
Intercept	Fatal injury rates	0.559	10.145	0.796
	Non-fatal injury rates	0.031	0.255	0.073
OSH inspectors	Fatal injury rates	0.274	3.016	0.334
	Non-fatal injury rates	0.430	6.026	0.578
Error	Fatal injury rates			
	Non-fatal injury rates			
Total	Fatal injury rates			
	Non-fatal injury rates			
Corrected total	Fatal injury rates			
	Non-fatal injury rates			

^a^R squared = 0.274 (adjusted R squared = 0.183). ^b^R squared = 0.430 (adjusted R squared = 0.358). ^c^Computed using alpha = 0.05.

**Table 7:** Parameter estimates.

Dependent variable	Parameter	*B*	Standard error	*t*	Significance	95% confidence interval ower bound
Fatal injury rates	Intercept	2.919	0.917	3.185	0.013	0.806
	OSH Inspectors	−1.588	0.915	−1.737	0.121	−3.698
Non-fatal injury rates	Intercept	−110.907	219.621	−0.505	0.627	−617.355
	OSH Inspectors	537.996	219.156	2.455	0.040	32.621

Dependent variable	Parameter	95% Confidence interval upper bound	Partial eta squared	Non-centrality parameter	Observed power^a^
Fatal injury rates	Intercept	5.033	0.559	3.185	0.796
	OSH inspectors	0.521	0.274	1.737	0.334
Non-fatal injury rates	Intercept	395.542	0.031	0.505	0.073
	OSH inspectors	1,043.372	0.430	2.455	0.578

^a^Computed using alpha = 0.05.

Thus, the authors performed a general multivariate regression analysis to examine if the OSH Inspectors to Employed Person per 10,000 ratios (IV) could predict SDG 8.1.1 (DV) or fatal injury rates (DV1) and non-fatal injury rates (DV2). Three major findings of the regression analysis were as follows:

- The relationship between the OSH inspectors to employed workers ratios and non-fatal injury rates in the region was significant, *F* (1, 8) = 6.026, *p* = 0.040 (*p* < 0.05), η^2^p = 0.430, observed power = 0.578. One practical implication of this result could be that a greater number of OSH inspectors may be required to increase business organizations’ compliance with OSH policies, and reports of more non-fatal workplace injuries in the regions may also increase.
- The relationship between OSH inspectors to employed workers ratios and fatal injury rates in the region was negative and not significant, F (1, 8) = 3.016, *p* = 0.121 (*p* > 0.05), η^2^p = 0.274, observed power = 0.334. One practical implication of this result could be that a greater number of OSH Inspectors might influence or decrease workplace fatalities in the regions. However, for greater reliability and credibility, future researchers may evaluate this assessment using larger sample sizes.
- Overall, this multivariate regression model was significant, *F* (2, 7) = 4.819, *p* = 0.048 (p < 0.05), η^2^p = 0.579, observed power = 0.597.

### 3.4 Limitations and further research

Regarding the limitations in the qualitative analysis of regional OSH policies, this review only explored the main OSH policies in North America and Southeast Asia. Any form of shortcoming where the contents are not adequately covered may be addressed in other forms, such as ministerial orders or directives made under those laws. Additionally, the authors used an unofficial English document from Thailand titled “Criteria for Hazardous Identification, Risk Assessment and Risk Management Plan B.E. 2000” as a part of regional OSH policies and risk management practice revision. Future researchers may need to refer to the official documents published in the regional language for the exact interpretation of the OSH policies. This report’s qualitative comparison of regional OSH policies differentiated the regional OSH policies mainly between performance-based and outcome-based policies and did not consider system-based OSH policy styles.

Regarding the limitations associated with quantitative analysis or general multivariate regression analysis, the authors used secondary self-reported data from the respective regions to the ILO for the quantitative analysis, and Saskatchewan’s data were also self-reported secondary data. The ILO’s statistics used in this research had significant missing datasets or errors and inconsistencies in how the data were collected or reported by the ILO’s member nations. Future researchers may review and, if required, differentiate the OSH performance dataset associated with the labor inspectors and OSH inspectors. In this review, the authors considered that regional workplace labor inspectors and OSH inspectors to be the same. This study was unable to compare the effectiveness of OSH policy and risk management practices between the North American Regions and the Southeast Asian Regions with two separate regression analyses: (1) the regression analysis of the North American regions datasets and (2) the regression analysis of the Southeast Asian regions (Table 8).

**Table 8:** Singapore’s annual OSH performance dataset used for regression analysis.

Years (2014–2023)	Independent variable	Dependent variable(s) (SDG 8.1.1 fatal and non-fatal injuries)	
	OSH inspectors (IV)	Fatal injury rates (DV1)	Non-fatal injury rates (DV2)
2023	1.19	0.99	622
2022	1.11	1.3	613
2021	1.2	1.1	386
2020	1	0.9	343
2019	0.95	1.1	395
2018	0.9	1.2	372
2017	0.88	1.2	368
2016	0.88	1.9	380
2015	0.91	1.9	362
2014	0.93	1.8	403

In this Singapore’s dataset, the OSH inspectors, also known as labor inspectors per 10,000 employed persons is independent variable (IV), and the SDG 8.1.1 is the dependent variable (DV), whereas fatal injuries per 100,000 workers is DV1 and non-fatal injuries per 100,000 is DV2 in the regression analysis. Adapted from reference (64).

Furthermore, Singapore was the only country that had complete datasets: the OSH inspectors to employed person ratios, fatal injury rates, and Non-fatal Injury rates. However, there were only nine annual datapoints for 9 years (2014–2023), while computing the regression analysis for Singapore. Therefore, the authors recommended that the readers be aware of the limitations pointed out in this report, including the limitations in the design of a multivariate regression study, the test of normality, and be cautious while generalizing or interpreting the findings of this investigation report. Future OSH researchers may utilize a larger (primary) dataset, or quarterly instead of annual datasets, to enhance credibility in the findings of the research.

This research also recommends exploring or examining the most effective or optimum ratios of regional OSH inspectors to employed person to exploit this ratio’s negative relationship with occupational non-fatal injury rates. OSH researchers may investigate to understand the relationship between the OSH officers, coordinators, or workplace labor inspectors and employed persons at any project, organizational level, or industry level in North America, Southeast Asia, or any other geographical regions. Stakeholders, be they regional regulatory bodies, individual business organizations, industries, or OSH professional bodies, may incorporate the OSH inspectors to employed persons ratios, but should continuously monitor the effectiveness of such interventions to ensure higher standards of safety and health compliance.

## 4 Conclusion

This comparative review between three North American and three Southeast Asian regions discovered that each region has its own national OSH policies. Although all those geographical regions shared a commitment to champion OSH risk management and damage prevention, the styles and methods of executing the OSH policies were different in terms of scope, criteria of compliance, and the level of comprehensiveness in detailing the requirements. The North American regions’ OSH policies were more performance-based, and the Southeast regions’ national OSH policies, influenced by the ILO’s OSH-MS, were more prescriptive OSH policies.

Based on the evidence observed during the qualitative analysis of the regional OSH policies and risk management practices, this review recommends that the OSH policymakers and practitioners in the Southeast Asian regions, and the North American regions consider regional or national OSH policies as just “living documents” and review those “living documents” more frequently and periodically in today’s agile and ever-changing high-risk business worlds for the sake of more effectively attaining global sustainable development goals: SDG 8.1.1.

Additionally, this research recommends that the ILO ensure that member nations follow a universal data collection and reporting methodology to prevent or minimize ambiguity or inconsistencies in the ILO’s publicly available statistics. Likewise, this research recommends that ILO’s member nations regularly record, report, and monitor OSH performance indicators, such as SDG 8.1.1, for continuous improvement of OSH performance.

Finally, this research encourages regional and global OSH policymakers, practitioners, and OSH academia to collaborate proactively, crafting, implementing, and promoting OSH policies based on evidence from the literature and job sites so that today’s stakeholders can ensure safer and healthier workplaces for tomorrow’s stakeholders. Thus, this study conducted a high-level comparison of OSH risk management policy styles between North America and Southeast Asia, making a scholarly attempt to explore the answers to three major questions of this review study. This review study identified the effectiveness of the regional OSH policies based on the secondary data, including workplace fatal and non-fatal injuries, as well as the OSH inspectors to employed person ratios. Moreover, this study offered evidence-based recommendations, including a conceptual framework (see Figure 3) for a regression study to enhance the OSH performance of SDG 8.1.1 regionally as well as globally.

## Data Availability

The data used in this study is publicly available. Additionally, for a list of regional OSH policies reviewed, see Figure 1 and for the quantitative datasets used in this review, see Tables 2 and 8.
